# Person-Organization Commitment: Bonds of Internal Consumer in the Context of Non-profit Organizations

**DOI:** 10.3389/fpsyg.2017.01227

**Published:** 2017-07-20

**Authors:** Emma Juaneda-Ayensa, Mónica Clavel San Emeterio, Carlos González-Menorca

**Affiliations:** ^1^Departamento de Economía y Empresa, Universidad de La Rioja Logroño, Spain; ^2^Facultad de Ciencias Jurídicas, Sociales y Humanidades, Universidad Internacional de La Rioja (UNIR) Logroño, Spain

**Keywords:** non-profit organizations, affective commitment, continuity commitment, normative commitment, confirmatory factor analysis

## Abstract

From an Organizational Behavior perspective, it is important to recognize the links generated between individuals and the organization that encourage a desire for permanence. After more than a half century of research, Organizational Commitment remains one of the open questions in the Psychology of Organizations. It is considered an essential factor for explaining individual behavior in the organization such as satisfaction, turnover intention, or loyalty. In this paper, we analyze different contributions regarding the nature of the bond between the individual and the organization. Taking into account the peculiarities of Non-profit Organizations, we present different interpretation for later validation, comparing results from the Confirmatory Factor Analysis of the four models obtained using exploratory factor analysis, both conducted on a sample of 235 members of Non-profit Organizations.

## Introduction

Uncertain and changing environments characterize the context which organizations have to develop their activity currently. In order to face such difficulties, one of the key aspects to win competitive advantage is to ensure that employees are committed, that they identify with the organization and accept its values and objectives as reflecting their own interests. Staff members of an organization prove fundamental to its success, especially where their satisfaction impacts on client satisfaction levels, it is necessary for organizations to perceive their employees as their first clients (Alves et al., 2015). Internal marketing should take corresponding priority over any external marketing processes (Ahmed and Rafiq, 1995; Flipo, 2007; Alves et al., 2015).

Organizational commitment has been highlighted as the primary attitudinal variable in the development of volunteer commitment and long-term retention (Stirling et al., 2011; Vecina et al., 2012) which are held to provide motivation (McCormick and Donohue, 2016). Although, there are few works, the obtained results in the frame of the Third Sector show differences with regard to the general multidimensional model as defined by Meyer and Allen (1984, 1991). One of the reason to consider is the differences between the organizations that operate across the sectors. The first is an important difference in the defining source of revenues.

The study of Commitment began with sociological theories that analyzed the impact of penalty systems on socially accepted values (Becker, 1960). But the work of Porter et al. (1974), which takes a sociological and psychological approach, was probably the origin of the study of links between the individual and the organization from the perspective of organizational behavior. Several decades later, Organizational Commitment is a complex concept that continues to be actively researched (Meyer et al., 2002, 2004; Allen, 2003; Cohen, 2003, 2007; Wasti, 2005; Ashman and Wintanley, 2006; Bergman, 2006; González and Guillén, 2008; Iqbal, 2010; Walumbwa et al., 2010; Stazyk et al., 2011; Klein et al., 2014; Reevy and Deason, 2014; Zayas-Ortiz et al., 2015; Hansen and Kjeldsen, 2017; Idris and Manganaro, 2017; Jaros, 2017; Wang et al., 2017) in attempting to define the relationships established between a person and the organization in which he/she works, due to the importance of the construct for employees and employers (Yousef, 2017). Although recently new approaches have appeared (e.g., Klein et al., 2014), the majority of researchers agree that organizational commitment should be treated as a multidimensional construct (Back et al., 2011) and that consistent correlations with other concepts vary with respect to dimensions. Notwithstanding, due to the use of different measurement scales and results regarding the internal structure there is no consensus regarding their interpretation hence the debate is still open (e.g., González and Guillén, 2008; Klein et al., 2014; Jaros, 2017).

Due to the aforementioned lack of consensus and the different results found in the body of literature regarding the Third Sector, the aim of this study is to gain an in-depth understanding of the internal structure of Organizational Commitment for Non-profit Organizations (NPOs), in order to understand the reason why people become involved in an organization and the types of bonds established between people and the organization. Considering the different models and theories concerning commitment, this work compares four structural models and the present paper is structured as follows. The first section reviews the main theories relating to organizational commitment in the body of scientific literature. Subsequently, we present our definition of commitment with different dimensions that are adapted to the field of NPOs for further analysis. Then, we present the results of the structural analysis of dimensions. Finally, the last section presents the main conclusions, taking into account the limitations of the study and the different implications for human resources management for NPOs.

## Literature review

### Organizational commitment: an open debate about the nature of the bond

During recent decades, Commitment has been defined and measured in different ways (Gupta, 2017). However, the lack of consensus regarding its definition does not imply the lack of a common body of knowledge that allows us to “distinguish it from other related constructs, such as satisfaction, motivation, implication” (Liou and Nyhan, 1994, p. 100).

Organizational commitment is defined as an emotional, moral and rational phenomenon (Ahmad and Oranye, 2010). Taking into account the different connotations as to the origin of the bond and the main differences among the different contributions, it can be seen in Table 1 that all the authors agree that commitment is a link with the organization that involves either behavior or attitude.

**Table 1:** Definitions of the commitment dimensions.

**Mental attitude**	**Dimension terminology and previous literature associated**	**Behavior associated**
Affective bonds	Affective (Meyer and Allen, 1984, 1991; Jaros et al., 1993)	All the authors agree it indicates a desire to stay in the organization and follow a course of action to achieve the organizational objectives and goals.
	Value (Angle and Perry, 1981; Mayer and Schoorman, 1992, 1998)	
	Moral (Penley and Gould, 1988; Jaros et al., 1993)	
	Normative (Cadwell et al., 1990; O'Reilly et al., 1991)	
Continuity bonds	Continuance (Mayer and Schoorman, 1992, 1998; Jaros et al., 1993).	Course of action that can be maintained to avoid the costs associated with leaving the organization or the perceived lack of alternatives.
	Alienative (Penley and Gould, 1988)	
Normative bonds	Normative (Wiener, 1982; Meyer and Allen, 1984, 1991; Meyer et al., 1993)	Perceived obligation to stay. No information about the behavior generated. Underdeveloped.

Therefore, it is necessary that the link involves particular behaviors or a positive attitude toward an organization that predisposes the individual to benefit the organization (Meyer and Herscovitch, 2001). Organizational commitment is the extent to which individuals psychologically identify with their work organizations (Idris and Manganaro, 2017). The nature of the links can vary and they include desire, perceived cost and obligation to continue a course of action (Table 1). Although it is useful to consider commitment as spread over a range from the emotional to the instrumental perspective, these approaches reflect different underlying components of commitment and therefore scales of measurement for explaining the construct dimensions will continue to be developed. But, the generally accepted feeling seems to indicate that the consequences of the multidimensional construct links lead us to links of different individuals. These types of links could be set as follows: (1) Affects or an affective link as an affective feeling or emotional link; (2) Fear or a repressive link as a feeling of being trapped; and (3) Normative links as feelings of obligation.

The studies relating to organizational commitment from an empirical point of view and the different interpretations of the links require us to rethink their meanings, especially within the scope of third sector NPOs and particularly, considering that they have volunteer resources. NPOs do not have financial wherewithal to implement human resources policies for promoting involvement and motivation, either for workers or for volunteers (Pearce, 1993; Boezeman and Ellemers, 2008), hence they have to use other tools to attract and retain human resources in the organization. It can be one of the tools that human resources managers utilize in order to analyze employee identification with organizational goals, and loyalty linking employees to their workplace (Zayas-Ortiz et al., 2015). In the next section, taking into account the different contributions made and organizational characteristics, we present the various bonds considered for these organizations.

### Definition of commitment: dimension content

In general, there is a scientific consensus regarding this conceptual delimitation. But, it diminishes as far as the taxonomy of links is concerned, or the way in which individuals feel tied to the organization. There have been several attempts to classify organizational commitment (Ahmad and Oranye, 2010). After reviewing the different contributions in the body of literature, we raise the idea that the nature of the bond generates different dimensions that, as aforementioned, can be summarized as affections, fears and obligations.

Among the different methods recognized in the field of psychology, our revision of the presented literature indicates that the previous studies usually work with models having different numbers of dimensions (Table 2). To the best of our knowledge, previous studies collected at least six meanings that are viewed as relevant to the level of effort demanded of the individual and his/her own acceptance (attitudinal aspects), and relate this and other factors of individual motivation with a decrease in probability of abandoning the organization. For that reason, exploration of the nature of the relationship and motivations of the individual become the fundamental factors. Next, we present the factors considered and justification for the most relevant factors selected.

**Table 2:** Dimensions of the Construct Organizational Commitment.

**Model**	**Dimensions**	**Empirical application**
One dimension	Affective (Porter et al., 1974)	Mowday et al., 1979; Morris and Sherman, 1981; Angle and Perry, 1983; Stumpf and Hartman, 1984; Lincoln and Kalleberg, 1985; Curry et al., 1986; Johnston et al., 1987; Pierce and Dunham, 1987; Brooke et al., 1988; Michaels et al., 1988; Sager and Johnston, 1989; Tett and Meyer, 1993; Baker and Baker, 1999; Dávila De León and Chacón Fuentes, 2003; Escrig et al., 2003; Scholarios and Marks, 2004; Reevy and Deason, 2014; Pignata et al., 2016; Chordiya et al., 2017; Hansen and Kjeldsen, 2017; Tremblay et al., 2017
Double dimension	Affective and continuous	Meyer and Allen, 1984; Mathieu and Zajac, 1990; Bayona et al., 1999; Brooks and Zeitz, 1999
	Value commitment and commitment to stay	Angle and Perry, 1981; Mayer and Schoorman, 1992, 1998
	Affective and continuance	Yilmaz, 2008
Triple dimension	Affective, continuance, and normative	Meyer et al., 1989; Allen and Meyer, 1990; Jaros et al., 1993; Dunham et al., 1994; Liou and Nyhan, 1994; De Frutos et al., 1998; Iverson and Buttigieg, 1999; Liao-Troth, 2001; Henkin and Marchiori, 2003; Powell and Meyer, 2004; Vandenberghe et al., 2004; Dawley et al., 2005; Boezeman and Ellemers, 2008; Wasti and Can, 2008; Fu et al., 2009; Ahmad and Oranye, 2010; Back et al., 2011; Top et al., 2015; Chiang and Liu, 2017; Tekingündüz et al., 2017
	Commitment to compliance, identification, and internalization	O'Reilly and Chatman, 1986; Harris et al., 1993
	Affective commitment, moral, and continuous	Jaros et al., 1993; Clugston, 2000; Tanner, 2007
	Moral commitment, calculative, and alienative	Penley and Gould, 1988

### Affective bonds. affections

In this first interpretation of commitment, we aim to gather the attitudes of an individual that link him/her to the organization, either because he/she appreciates the entity values, or because he/she identifies with them. It has been identified as an antecedent of organization citizenship behavior (Wang et al., 2017). O'Reilly and Chatman (1986) show that this type of attitude arises when workers behave in a certain way, because they want to remain in the organization due to its attractiveness (values and goals), even though its values may not be those that the person would adopt. The individual accepts the influence of values to establish or maintain a satisfactory relationship, hence this dimension emphasizes an aspect of socialization of an instrumental nature. The affection is shown by a feeling of pride that is generated outwards toward the reference group, which finally generates self-esteem owing to the sense of belonging within the organization. Feelings of pride and respect are seen as important motivators in the field of voluntary organizations (Boezeman and Ellemers, 2008). The type of commitment (compared to continuance and normative commitment) that is expected to be most clearly related both to organizational issues would be for example attendance, performance, and organizational citizenship behavior (Hansen and Kjeldsen, 2017) or dimensions of attitudes toward organizational change (Yousef, 2017). The most used and validated measure of organizational commitment in the body of public management literature (Hansen and Kjeldsen, 2017) is affective commitment, and it is especially relevant to volunteering, given the intrinsic motivation, non-monetizable, socio-emotional need of fulfillment and positive work experience ascribed to voluntary contributions to an organization of time, energy and expertise (Ohana and Meyer, 2016).

Although, non-profit employees often feel they are underpaid (Light, 2003; Kim and Lee, 2007; Handy et al., 2008), they may be willing to sacrifice some money (from wage, income) in order to serve a cause or specific social mission (Ohana and Meyer, 2016). In this way, the organization's affective commitment is important in a context of scarce financial resources and can help to resolve the dilemma faced by non-profit managers of how to keep employees committed without offering them the highest possible salary (Ohana and Meyer, 2016).

Secondly, and still referring to this dimension, other studies show an individual's identification with the value system of the organization. In this case, the identification reflects a behavior that is supported by internal values and goals which are also connected with those adopted by the organization. In this case, the concept involves more than mere loyalty. It implies an active relationship with the organization in reaching its goals, as a way to serve one's own interests. In this regard, we will adopt the meaning of Affective from the model of Meyer and Allen (1984, 1991), the significance of the definition given by Mowday et al. (1979), the Internalization dimension of O'Reilly and Chatman (1986), and the meaning compiled under Moral Commitment from Jaros et al. (1993).

The reason why we question the breakdown into these two subdimensions is due to the diversity of interpretations of the emotional aspect of the term. This difference can be seen in the model developed by Jaros et al. (1993) under the itemization of the affective dimension and the meaning given to the moral dimension, or in the O'Reilly and Chatman model (1986) as comprised in the dimensions of identification and internalization.

Affective commitment has been linked to measured involvement in organizational activities, a strong willingness to contribute to achieving organizational goals and a strong desire to remain with the organization (Walumbwa et al., 2010; Idris and Manganaro, 2017). It is found to be strongly related to important organizational outcomes such as attendance, turnover, performance, and organizational citizenship behavior, as well as individual outcomes such as stress and work family conflict (Meyer et al., 2002; Stazyk et al., 2011; Chordiya et al., 2017).

### Continuity bonds. fears

The second attitudinal dimension explains the relationship between an individual and the organization as a sense that his/her withdrawal would imply the loss of some acquired conditions or rights, or that he/she has no other labor alternative (Tekingündüz et al., 2017). According to the definition by Becker (1960), organizational commitment is associated with the assessment made by an individual of the costs involved in the abandonment of the organization where he/she works and/or the costs of renouncing a situation or status resulting from his/her efforts. Commitment is thus defined as a willingness to deploy a determined consistent line of behavior as a result of the accumulation of investments that could be lost if that line of action was abandoned (González and Antón, 1995). Consequently, in the light of commitment related to the investments made, all actions performed by an individual after becoming part of an organization will lead to attempting to justify his/her continuance (Becker, 1960; Salancik, 1977).

Along with the prior aspects, the explanation based on the continuity/fear argument, another key factor stands out: the lack of alternatives. McGee and Ford (1987) pioneered the study of commitment bi-dimensionality based on Becker's theory. These authors suggested the existence of two interpretable factors: the perceived sacrifice associated with neglect and the lack of alternatives.

The results obtained that split Continuous commitment into two dimensions are supported by studies that use discriminatory factor analysis, such as studies by Allen and Meyer (1996), Hackett et al. (1994), Iverson and Buttigieg (1999), Meyer et al. (2002), Meyer et al. (1990), and Somers (1993). Although there are other results that indicate uni-dimensionality such as those results obtained by Dunham et al. (1994), Kou et al. (1997), and Powell and Meyer (2004).

Moreover, works in the field of voluntary organizations demonstrate empirically that normative commitment does not imply a significant relationship for this type of person-organization relationships (Liao-Troth, 2001; Stephens et al., 2004; Dawley et al., 2005).

### Normative bonds. obligations

For this third and final dimension of reference, there is less research and empirical contributions in the body of literature than for those previously mentioned dimension, despite its importance in explaining Organizational Commitment. One of the main problems found in the definition of this dimension is the lack of consensus as to its meaning, although the work goes on to consider that the policy linkages reflect a sense of obligation (Meyer and Herscovitch, 2001). As such, a committed person will feel compelled to stay connected with the organization (normative linking). One of the factors that has contributed to the lack of clarity with respect to the affective dimension relates to the high levels of correlation with this dimension, as obtained in previous studies. The person has normative commitment while showing loyalty to their organization and they present suitable behavior and conduct with motivation for doing good for the organization (Tekingündüz et al., 2017). This dimension is still unknown and probably the most controversial dimension (Jaros, 2017) bearing in mind the implications included in previous works (Juaneda-Ayensa and González-Menorca, 2007; González and Guillén, 2008; Grant et al., 2008; Meyer and Parfyonova, 2010), we break this dimension into two types of link: Moral Obligation and Gratitude.

The need for internal consistency presented in the cognitive dissonance theory of Festinger (1957) is reflected in the concept of Moral Obligation. It describes the tendency of individuals to reconcile internal inconsistencies. Because of this desire for internal consistency, attitudes and beliefs of an individual may not only be determinants of their behavior, but a result of it. This approach assumes that attitudes are relatively private, malleable, and not always clearly identifiable. In comparison, behaviors are more public, and once acted out, irrevocable (although the consequences are not). Thus, attitudes, which are easily modifiable elements, will be molded around the least malleable factor, or behaviors, the mechanism by which the individual strives to maintain consistency between them (Oliver, 1990). This would mean that an action taken modifies an attitude if there is any inconsistency between them. According to this theory, people who are committed to participate/collaborate on a project should continue with it in order to avoid contradicting his/her line of action that was already begun. If they were to decide to interrupt it, it would be a contradiction and therefore internally inconsistent.

The second meaning of this dimension is Gratitude, defined as a sense of obligation due to the feeling of having received more than what has been given. In this case, social exchange theory is of significant importance. According to Blau (1964), relationships between two parties, when one of them provides benefits to the other, the imbalance in the relationship confers an obligation on the second party. The person feels there is an imbalance regarding benefit, and this fosters a sense of debt to the organization, i.e., strengthens the feeling of obligation to the organization and attempts to balance it, believing this behavior to be appropriate to contribute to balance in the exchange.

We believe it is important to look at this dimension in depth in the context of NPOs. In an attempt to enhance the theory of normative commitment, González and Guillén (2008) consider that the normative dimension should be grouped with rational judgments regarding the moral sphere of the individual, and include whatever has to do with moral judgments (fairness) and moral practice (responsibility), which to some extent is what we intend to compile under the dimensions of Gratitude and Moral Obligation. On the other hand, we consider that in the NPO, this moral connotation is relevant and it is particularly influenced by NPO characteristics, including the role of demands and political pressure aimed at ensuring a level of rights for a group or society in general and the role these activities play in transforming individuals. From this point of view, linking to one of these organizations is a public demonstration of certain convictions, and activities performed under the auspices of the entity support identification of the individual with the organization's ideology. In the event that there is incomplete identification between the value and belief systems of the organization and the individual, the size of the organization, and the difficulty to model this value system will indoctrinate individuals as a tool of socialization. The result will be a link with the organization as a consistent behavior based on public demonstration of an ideological system. However, we must include ideological or ethical issues in this section [the significance of the normative commitment of the Allen and Meyer (1990) scale] in which the relationship is reflected and manifested in the permanence within the organization that are generated from the value system of the individual who becomes “tied” to the acquired commitments.

When gratitude is felt toward the organization, the more relevant argument is based on the fact that most of these organizations provide services that are not provided by the public or private sector, and the organizations offer solutions to problems for people in situations of some complexity, helping them feel connected to the organization as a show of gratitude for the support received.

In addition, this type of link may have a particular impact on NPOs and as suggested by Meyer and Allen (1997), commitment characterized by a sense of obligation might be a better predictor of employment outcomes in collective contexts where social ties and regulatory obligations are most relevant.

Then, we propose a concept as a three-dimensional model which, depending on the different connotations attributed by the authors, can have each of its dimensions broken down into two sub-dimensions (Figure 2).

Affective commitment has been linked to increased involvement in organizational activities, involvement in organizational activities, a strong willingness to contribute to achieving organizational goals and a strong desire to remain with the organization (Walumbwa et al., 2010; Idris and Manganaro, 2017). Continuance and normative dimensions of commitment have been critiqued for their inconsistencies with affective commitment (Chordiya et al., 2017). Normative commitment is usually strongly linked to affective commitment (Guerrero and Herrbach, 2009) and is linked to individuals' sense of obligation to stay in the organization (Wang et al., 2017).

## Materials and methods

As stated previously, there is a question regarding the link between the individual and the organization and, although most empirical studies are based on the one-dimensional Porter et al. (1974) model or the three-dimensional Allen and Meyer (1990) model, in our case we decided to rethink the links and try to show the existence of links that have been overlooked or have had lesser attention in the body of literature on organizational commitment. The main reason for this decision was that this study was undertaken in a particular environment within which the relationships between members of the organization and the organization itself showed the characteristics we have already mentioned. Because of the complexity of the construction and due to the lack of works in the Spanish language that are adapted to Third Sector organizations, we decided to develop an *ad hoc* measurement tool that would allow us to achieve our goals. Hence, we developed a measurement scale based on the main previous works, in order to obtain a reliable measurement tool that would enable us to assess most accurately the level of commitment to an organization. First, we defined the dimensions and later allocated the items (Table 3) to the different connotations set out. We obtained a 20-item scale from experimental analysis, although we decided to add a control item that enabled us to gather the perceptions of individuals regarding their level of commitment to the organization (Sánchez and Sarabia, 1999). We used a Likert scale of 0–10 for measurement.

**Table 3:** Definition of organizational commitment scale.

**ITEM**	
1. I am deeply committed to this organization	Control variable Sources
2. I am proud to belong to this organization.	OCQ
3. This organization is a good place to work.	OCQ
4. I like people in my social environment to know that I participate in this organization.	OCQ, AMM
5. When I talk to my acquaintances about the organization I convey favorable information about it.	OCQ
6. I positively assess the goals the organization has.	OCQ, AMM
7. I identify with the values that are promoted by the organization.	OCQ
8. I am concerned about the future of the organization.	OCQ
9. I feel the problems of the organization as my owns.	AMM
10. I have made a great effort for this organization.	Jaros et al., 1993
11. If I left the organization, my personal situation would get worse.	OCQ, AMM
12. Working in this organization is better than working in another one with similar activities.	OCQ
13. I have a lot to lose if I leave the organization.	AMM, Meyer and Herscovitch (2001)
14. It would be difficult to carry out my work in another organization.	AMM
15. It would be very difficult to find an alternative activity if I had to leave the organization.	AMM
16. I receive more from the organization than I give.	Grant et al., 2008; Meyer and Parfyonova, 2010
17. I am indebted for what the organization has done for me.	
18. I feel morally obliged to continue in this organization.	AMM
19. I am loyal to the organization.	OCQ, AMM
20. My conscience compels me to continue in the organization.	AMM
21. Remaining with this organization is consistent with my way of thinking.	AMM

*OCQ, Organizational Commitment Questionnaire (Mowday et al., 1979). AMM, Allen and Meyer Measurement (Allen and Meyer, 1990)*.

The analysis was performed using IBM SPSS 24 for the exploratory factor analysis and using AMOS 24 for the confirmatory factor analysis. Validation of the tool was undertaken by following considerations provided by work on testing sociometric properties required by the scales of measure, an issue that enjoys broad endorsement in the body of marketing and organization literature (Lévy and Varela, 2006; Camisón and Cruz, 2008; Hair et al., 2010).

Exploratory factor analysis was applied to the principal axis factoring method with Varimax rotation in order to compare the underlying structure of empirical data with the theoretical structure of the resulting models from the literature review and which are presented in the above. In the first extraction, the result obtained for the analyzed sample was five dimensions, which were automatically determined. But, as we wanted to test the adequacy with respect to different theoretical proposals, we decided to obtain the factor analysis for models with 3, 4, and 6 dimensions (Table 4). Following the recommendations of Hair (Hair et al., 2010) to develop the most suitable, commitment model, and in order to facilitate our work on the confirmatory factor analysis, we proposed comparisons among the four structures with 3, 4, 5, and 6 latent dimensions as a rigorousness test, by comparing competing models (Bentler and Bonett, 1980; Hair et al., 2010).

**Table 4:** Definition of dimensions.

**Item**	**5 Dimensions model**		**3 Dimensions model**		**4 Dimensions model**		**6 Dimensions model**	
2	1	Identification	1	Affective	1	Affective	1	Identification
3	1		1		1		1	
4	1		1		1		1	
5	1		1		1		1	
19	1		1		1		1	
10	2	Continuance	2	Continuance	2	Continuance	5	Cost of leaves
11	2		2		2		3	Lack of alternatives
12	2		2		2		3	
13	2		2		2		5	Cost of leaves
14	2		2		2		3	Lack of Alternatives
15	2		2		2		3	
6	3	Internalization	1	Affective	1	Affective	2	Internalization
7	3		1		1		2	
8	3		1		1		2	
9	3		1		1		2	
18	4	Moral obligation	3	Normative	3	Moral obligation	4	Moral obligation
20	4		3		3		4	
21	4		3		3		4	
16	5	Gratitude	3		4	Gratitude	6	Gratitude
17	5		3		4		6	

Parameters (standardized factor loadings) for the confirmatory factor analysis construct elements were obtained through structural equation systems. To carry out the estimation of model parameters, we used the original data matrix instead of the correlation matrix as input, because of the information available to us and because our desire is to explain the nature of the latent construct. Moreover, taking into account the lack of multivariate normality, we decided to use the Asymptotically Distribution Free (ADF) method of estimating function—guided by the considerations presented by Hair et al. (2010). One of the main drawbacks of the method is its higher demand with regard to sample size (Hair et al., 2010). In order to minimize the number of model parameters and to increase the degrees of freedom, we decided to group the observed measures of the same latent variable, the dimensions of commitment, in a composite score, the arithmetic mean, depending on the results of the exploratory factor analysis.

### Sample

The information was obtained from 14 non-profit organizations that operate in various fields of activity, but all of them are characterized as direct services. The areas of activity are services for children, mental disability, and the promotion of employment, physical disability, and a volunteer organization. The samples were obtained by the directors of the entities. The directors distributed the questionnaires and they committed themselves to respecting the anonymity of those respondents wishing to undertake the completion of the questionnaires.

The final sample consists of 235 questionnaires, of which 156 (66.38%) pertain to workers and 79 (33.61%) pertain to volunteers. The average seniority in the organization is 4.51 years with a standard deviation of 4.67 and a maximum of 26 years and a minimum of 6 months. Sixteen percent of the sample consisted of people who stayed less than a year in the organization, 46.6% were there for a period of 1–5 years, 27.7% for 5–10 years, and only 9.7% spent over 10 years in the organization. As for the level of education, the largest proportion of the sample comprised people with a mid-level university education (34.7%) followed by graduate degrees (25.4%), and only 10.9% have a level of basic studies.

## Results

**Figure 1:**
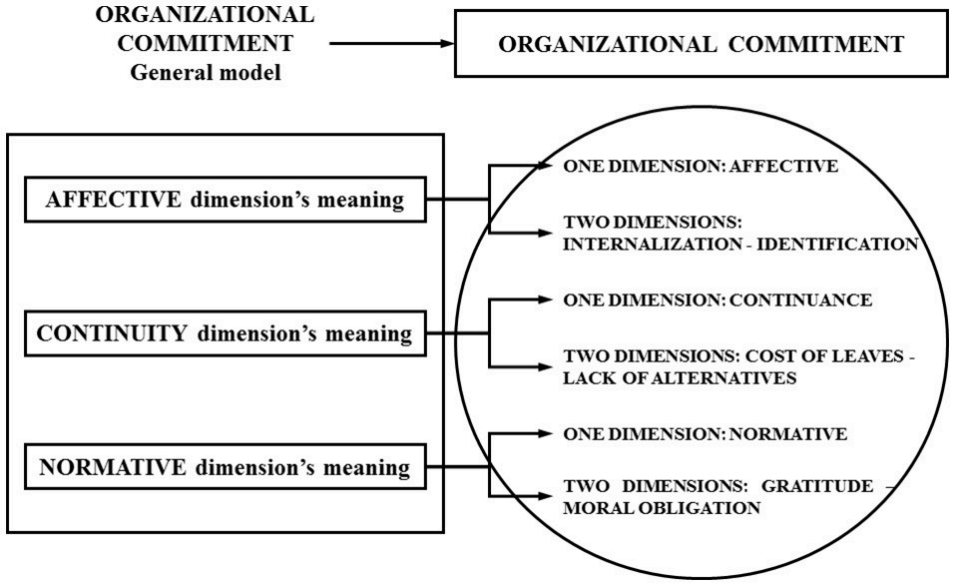
Organizational Commitment: dimensions debate.

The first step in the analysis was the validation of the measurement tool for the configuration proposed in Figure 1. The Bartlett sphericity test (χ^2^ = 2828.781; *p* < 0.001) and the Kaiser–Meyer–Olkin statistic (KMO = 0.861) report that the matrix of correlations for the exploratory factorial analysis factors proves to be very good, hence, appropriate to describing the data structure (*p* < 0.001; KMO > 0.8; Hair et al., 2010; Lévy and Varela, 2006; Camisón and Cruz, 2008). For reliability and validity of the scale, we calculated Cronbach's α of the composite reliability and the extracted variance (Camisón and Cruz, 2008). Cronbach's α-value obtained was 0.906 (Cronbach's α > 0.7; Lévy and Varela, 2006; Camisón and Cruz, 2008; Hair et al., 2010), which is high enough to believe that our scale is reliable and the factors account for 59.6% (3 dimensions), 65.9% (4 dimensions), 71.7% (5 dimensions), and 75.8% (6 dimensions) of the variance in the original data.

**Figure 2:**
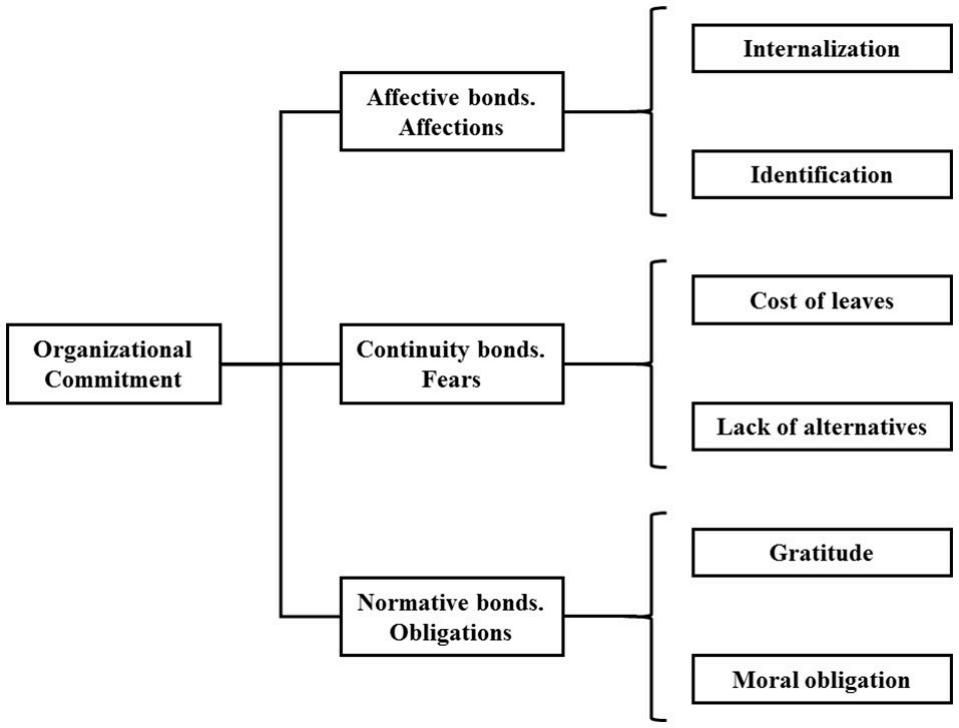
Commitment model in NPO.

Once it was verified that the requirements for using factor analysis (Hair et al., 2010) were fulfilled (Table 5), we performed exploratory factor analysis using the extraction method of Principal Component Analysis with scale items that reflected the different connotations regarding the individual-organization links as previously defined.

**Table 5:** Exploratory factor analysis.

**KAISER-MEYER-OLKIN MEASURE OF SAMPLING ADEQUACY AND BARTLETT'S TEST OF SPHERICITY**			
α		0.906	
Items (Number)		20	
KMO Index		0.861	
Bartlett Sphericity text	Chi-Square aprox.	2828.781	
	Degree of Freedom	190	
	Significativy	0.000	
**CUMULATIVE PROPORTION OF TOTAL VARIANCE**			
**3 Dimensions model**	**4 Dimensions model**	**5 Dimensions model**	**6 Dimensions model**
59.624%	65.888%	71.684%	75.763%

Exploratory factor analysis showed us the different configurations in models of 3, 4, 5, and 6 dimensions (Table 6).

**Table 6:** Rotated component matrix.

**3 Dimensions model**	**1**	**2**	**3**			
α	0.908	0.838	0.822			
Item 2. I am proud to belong to this organization.	**0.813**	0.153	0.224			
Item 5. When I talk…I convey favorable information about it.	**0.772**	0.166	0.260			
Item 7. I identify with the values that are promoted…	**0.768**	−0.024	0.202			
Item 4. I like people in my social …I participate in this organization	**0.742**	0.233	0.134			
Item 8. I am concerned about the future of the organization.	**0.741**	0.037	0.090			
Item 6. I positively assess the goals the organization has.	**0.732**	−0.058	0.183			
Item 3. This organization is a good place to work.	**0.721**	0.195	0.194			
Item 19. I am loyal to the organization.	**0.689**	0.218	0.244			
Item 9. I feel the problems of the organization as my owns.	**0.657**	0.154	0.174			
Item 13. I have a lot to lose if I leave the organization.	0.200	**0.814**	0.020			
Item 11. If I left …my personal situation would get worse.	0.066	**0.752**	0.161			
Item 12. Working in this organization …one with similar activities.	0.309	**0.724**	0.070			
Item 15. It would be very difficult to find an alternative activity …	−0.125	**0.713**	0.397			
Item 14. It would be difficult to carry out my work …	−0.059	**0.636**	0.426			
Item 10. I have made a great effort for this organization.	0.349	**0.590**	−0.115			
Item 18. I feel morally obliged to continue in this organization.	0.282	0.154	**0.743**			
Item 17. I am indebted for what the organization has done for me.	0.192	0.045	**0.740**			
Item 20. My conscience compels me to continue in the organization.	0.223	0.154	**0.727**			
Item 16. I receive more from the organization than I give.	0.304	0.110	**0.661**			
Item 21. Remaining is consistent with my way of thinking.	0.468	0.167	**0.516**			
**4 Dimensions model**	**1**	**2**	**3**	**4**		
α	0.908	0.838	0.847	0.698		
Item 2. I am proud to belong to this organization.	**0.811**	0.166	0.201	0.111		
Item 7. I identify with the values that are promoted…	**0.800**	−0.019	0.048	0.209		
Item 5. When I talk…I convey favorable information about it.	**0.765**	0.176	0.248	0.119		
Item 6. I positively assess the goals the organization has.	**0.765**	−0.052	0.030	0.197		
Item 8. I am concerned about the future of the organization.	**0.758**	0.052	0.022	0.086		
Item 4. I like people in my social …I participate in this organization.	**0.710**	0.255	0.251	−0.049		
Item 3. This organization is a good place to work.	**0.698**	0.210	0.262	0.021		
Item 19. I am loyal to the organization.	**0.659**	0.231	0.326	0.032		
Item 9. I feel the problems of the organization as my owns.	**0.657**	0.164	0.146	0.099		
Item 13. I have a lot to lose if I leave the organization.	0.149	**0.823**	0.160	−0.052		
esc 11. If I left …my personal situation would get worse.	0.052	**0.740**	0.104	0.192		
esc 12. Working in this organization …one with similar activities.	0.284	**0.728**	0.094	0.067		
esc 15. It would be very difficult to find an alternative activity…	−0.10	**0.670**	0.113	0.506		
Item 10. I have made a great effort for this organization.	0.304	**0.614**	0.063	−0.169		
Item 20. My conscience compels me to continue in the organization.	0.151	0.133	**0.882**	0.178		
Item 18. I feel morally obliged to continue in this organization.	0.228	0.129	**0.814**	0.262		
Item 21 Remaining is consistent with my way of thinking.	0.403	0.165	**0.693**	0.062		
Item 17. I am indebted for what the organization has done for me.	0.263	−0.012	0.259	**0.767**		
Item 16. I receive more from the organization than I give.	0.368	0.062	0.224	**0.695**		
Item 14. It would be difficult to carry out my work …	−0.007	0.588	0.022	**0.621**		
**5 Dimensions model**	**1**	**2**	**3**	**4**	**5**	
α	0.901	0.838	0.841	0.847	0.698	
Item 3. This organization is a good place to work.	**0.793**	0.144	0.207	0.148	0.108	
Item 4. I like people in my social …I participate in this organization	**0.783**	0.185	0.242	0.147	0.027	
Item 5. When I talk…I convey favorable information about it.	**0.762**	0.126	0.326	0.157	0.194	
Item 19. I am loyal to the organization.	**0.707**	0.176	0.243	0.238	0.095	
Item 2. I am proud to belong to this organization.	**0.706**	0.125	0.446	0.140	0.165	
Item 13. I have a lot to lose if I leave the organization.	0.204	**0.804**	0.067	0.154	−0.119	
Item 11. If I left …my personal situation would get worse.	0.166	**0.739**	−0.056	0.077	0.146	
Item 15. It would be very difficult to find an alternative activity…	−0.092	**0.724**	−0.034	0.136	0.423	
Item 12 Working in this organization …one with similar activities.	0.356	**0.704**	0.087	0.055	0.038	
Item 14. It would be difficult to carry out my work …	−0.098	**0.657**	0.091	0.059	0.537	
Item 10. I have made a great effort for this organization.	0.147	**0.601**	0.336	0.107	−0.252	
Item 8. I am concerned about the future of the organization.	0.255	0.070	**0.820**	0.105	0.035	
Item 9. I feel the problems of the organization as my owns.	0.182	0.188	**0.763**	0.240	0.025	
Item 7. I identify with the values that are promoted…	0.412	−0.011	**0.709**	0.077	0.207	
Item 6. I positively assess the goals the organization has.	0.362	−0.041	**0.707**	0.068	0.191	
Item 20 My conscience compels me to continue in the organization.	0.144	0.144	0.102	**0.898**	0.153	
Item 18. I feel morally obliged to continue in this organization.	0.167	0.148	0.180	**0.834**	0.236	
Item 21 Remaining is consistent with my way of thinking.	0.422	0.140	0.178	**0.662**	0.078	
Item 17. I am indebted for what the organization has done for me.	0.177	0.045	0.156	0.242	**0.786**	
Item 16. I receive more from the organization than I give.	0.331	0.098	0.157	0.178	**0.733**	
**6 Dimensions model**	**1**	**2**	**3**	**4**	**5**	**6**
α	0.901	0.841	0.814	0.847	0.582	0.698
Item 3. This organization is a good place to work.	**0.825**	0.218	0.124	0.148	0.025	0.068
Item 4. I like people in my social …I participate in this organization	**0.759**	0.210	−0.021	0.134	0.267	0.149
Item 5. When I talk…I convey favorable information about it.	**0.745**	0.305	0.029	0.148	0.146	0.270
Item 19. I am loyal to the organization.	**0.739**	0.251	0.142	0.238	0.061	0.058
Item 2 2. I am proud to belong to this organization.	**0.696**	0.429	0.033	0.132	0.144	0.229
Item 8. I am concerned about the future of the organization.	0.290	**0.836**	0.083	0.107	0.019	−0.011
Item 9. I feel the problems of the organization as my owns.	0.225	**0.776**	0.175	0.244	0.094	−0.040
Item 7. I identify with the values that are promoted…	0.374	**0.682**	−0.078	0.067	0.116	0.323
Item 6. I positively assess the goals the organization has.	0.316	**0.676**	−0.119	0.056	0.122	0.328
Item 15. It would be very difficult to find an alternative activity.	0.014	−0.003	**0.876**	0.155	0.130	0.107
Item 14. It would be difficult to carry out my work.	−0.006	0.118	**0.850**	0.077	0.087	0.238
Item 11. If I left …my personal situation would get worse.	0.196	−0.084	**0.559**	0.077	0.485	0.089
Item 12. Working in this organization …one with similar activities.	0.406	0.073	**0.520**	0.057	0.443	−0.041
Item 20. My conscience compels me to continue in the organization.	0.145	0.093	0.107	**0.896**	0.110	0.163
Item 18. I feel morally obliged to continue in this organization.	0.157	0.163	0.114	**0.830**	0.127	0.265
Item 21. Remaining is consistent with my way of thinking.	0.455	0.189	0.129	**0.664**	0.037	0.031
Item 10. I have made a great effort for this organization.	0.080	0.243	0.057	0.084	**0.849**	0.018
Item 13. I have a lot to lose if I leave the organization.	0.195	0.003	0.380	0.142	**0.769**	−0.019
Item 17. I am indebted for what the organization has done for me.	0.122	0.126	0.198	0.235	−0.030	**0.825**
Item 16. I receive more from the organization than I give.	0.278	0.123	0.200	0.170	0.027	**0.784**

*The bold values mean the factor loads on the factor to which they are assigned after the factorial analysis performed*.

Once the factors were extracted and the models to be evaluated were defined, the confirmatory factor analysis was conducted. Before examining the estimated parameters, model adjustments were checked (Table 7).

**Table 7:** Goodness-of-fit of alternative models of organizational commitment.

**Model**		**3 Dimensions**	**4 Dimensions**	**5 Dimensions**	**6 Dimensions**
Reliability	Composite reliability	0.716	0.736	0.785	0.845
	Variance explained	0.465	0.416	0.431	0.479
	α	0.920	0.920	0.920	.920
Absolut F	Chi-square	0	0.326	12.621	46.056
	df	0	2	5	9
	Sig		0.849	0.027	0
	Chi-square difference text^a^		0.326_−2_	12.295_−3_	33.435_−4_
	GFI	1	0.999	0.973	0.912
	RMSEA	0.305	0	0.081	0.133
Incremental	NFI	1	0.996	0.861	0.69
	TLI		1.072	0.811	0.538
	CFI	1	1	0.906	0.723
Parsimonia	AGFI		0.996	0.92	0.794
	Normalized Chi-square		0.163	2.524	0.117

a *The Chi-square text (p < 0.05) for df = 2 its value is 5.991; df = 3 is 7.815, and df = 4 is 9.488 (Malhotra, 2008)*.

As can be seen, the data show that all models have an adequate level of measurement reliability (composite reliability > 0.7; AVE > 0.4; Cronbach's α > 0.7; Lévy and Varela, 2006; Camisón and Cruz, 2008; Hair et al., 2010). In relation to absolute fit, we can see that the 3-D model has a Chi-square value equal to 0, meaning that this would be the model that enables us to ensure the ability to reproduce the observed matrix, although this feature is not useful for us due to the difficult generalization of the models that were identified as having no degrees of freedom (Hair et al., 2010). The 4-D model also has quite a high level of global adjustment (GFI > 0.9; Chi-square sig > 0.05; Lévy and Varela, 2006; Hair et al., 2010), followed by the 5-D model which does not allow us to accept the null hypothesis of equality between the observed and reproduced matrices (Chi-square sig < 0.05), although the value of the Chi-square standard indicator shows an acceptable value. The 3-D model provides a good fit although the RMSEA value behaves with values above those indicated as appropriate (RMSEA < 0.08; Hair et al., 2010; Lévy and Varela, 2006). The indicators of incremental and Parsimony adjustment (AGFI > 0.9 and 1 < Normalized Chi-square < 5; Hair et al., 2010; Lévy and Varela, 2006) of the 4-D model are the best (AGFI = 0.996; Normalized Chi-square = 0.163) followed by those of the 5-D (AGFI = 0.92; Normalized Chi-square = 2.524) model which are also acceptable. The 6-D model does not present a good fit in any of the analyzed adjustment dimensions.

In Table 8, the results of confirmatory factor analysis of the presented models can be seen. The 3-D model shows two main dimensions of organizational commitment, the size of links of Affective and Regulatory type (desire of belonging and sense of obligation), both of which have Standardized Regression Weights (SRW) = 0.795, while the continuous dimension (duty to stay) is not confirmed (SRW < 0.7; Lévy and Varela, 2006; Hair et al., 2010). This model is that which is usually considered and it has been compiled from the contributions of previous work (Allen and Meyer, 1990; Meyer et al., 2002). We aimed at collecting different interpretations offered in accordance with the context to apply, although in our case the results do not confirm the continuity dimension in the way they confirm the normative dimension. In the case of the 4-D model, the Moral Obligation (SRW = 0.723) and affection dimensions (SRW = 0.719) are confirmed, followed by values close to 0.7 of the Gratitude dimension (SRW = 0.616), and again the continuity dimension (SRW < 0.7; Lévy and Varela, 2006; Hair et al., 2010) does not show a high enough value. In the following model, continuing with the breakdown of the affective dimension, we find that both dimensions are confirmed, leaving all other dimensions with standard load values below the minimum (0.7), the Continuity Factor being that with the lower value (close to 0.5).

**Table 8:** Confirmatory factor analysis.

	**SRW**	***R*^2^**	**Errors**	**C.R**.	***P***
**3 DIMENSIONS MODEL**					
Affective	0.795	0.63	0.368	–	–
Continuance	0.498	0.25	0.752	5.404	^***^
Normative	0.795	0.63	0.368	5.992	^***^
**4 DIMENSIONS MODEL**					
Affective	0.719	0.52	0.483	–	–
Continuance	0.496	0.25	0.754	5.443	^***^
Moral obligation	0.723	0.52	0.477	7.865	^***^
Gratitude	0.616	0.38	0.621	7.542	^***^
**5 DIMENSIONS MODEL**					
Identification	0.855	0.73	0.269	–	–
Internalization	0.716	0.51	0.487	8.274	^***^
Moral obligation	0.617	0.38	0.619	8.215	^***^
Gratitude	0.538	0.29	0.711	7.319	^***^
Continuance	0.493	0.24	0.757	5.14	^***^
**6 DIMENSIONS MODEL**					
Identification	0.787	0.62	0.381	–	–
Internalization	0.679	0.46	0.539	8.114	^***^
Moral obligation	0.655	0.43	0.571	8.572	^***^
Gratitude	0.566	0.32	0.680	8.196	^***^
Lack of alternatives	0.728	0.53	0.470	7.733	^***^
Cost of leaves	0.716	0.51	0.487	7.67	^***^

*** *p < 0.001. C.R., Critical Ratio; SRW, Standardized Regression Weights*.

Finally, the 6-D model shows Commitment factors as the Pride dimension, followed by the Lack of Alternatives, the Costs of Abandonment and we could accept as confirmed both Identification (0.679) and Moral Obligation (0.655), although this model cannot be considered valid and replicable due to the adjustment problems it presents.

## Discussion

The results of this research make theoretical contributions to understanding the underlying nature of links between individuals and Non-profit Organizations (NPOs) and the reason why people (workers and volunteers) become involved in an organization, and managerial implications to improve the human resources management in organizations, especially in NPOs.

### Theoretical contributions

One of the main drawbacks in the organizational commitment study is the use of different measurement scales, and the problems inherent to cultural questions, and the linguistic adaptation of questionnaires to specific contexts. In this sense this work analyzes the meaning of the construct identifying different structures and comparing among them. From the comparative analysis of structural models, we can highlight several aspects.

First, in relation to the interpretation of the composition of the latent variable Organizational Commitment, it consists mainly of contributions from the individual's emotional bonds with respect to the organization, followed by normative and yet, the Continuity dimension values are not enough to ensure that this type of relationship creates a relevant link with respect to the entity. Therefore, we cannot accept the existence of the Continuity dimension as reflected in other studies (Meyer et al., 1990, 2002; Somers, 1993; Dunham et al., 1994; Hackett et al., 1994; Iverson and Buttigieg, 1999; Powell and Meyer, 2004), but it confirms, to the best of our knowledge, the results obtained in previous research relating to the scope of voluntary activities (Liao-Troth, 2001; Stephens et al., 2004; Dawley et al., 2005).

According to previous studies (i.e., Meyer and Allen, 1984, 1991; O'Reilly and Chatman, 1986; Jaros et al., 1993; Boezeman and Ellemers, 2008) the breakdown of the Affective dimension is confirmed and we obtain empirical evidence of the difference between the links generated by feelings of Identification and those generated by feelings of Pride of ownership.

The Commitment configuration to reflect upon is related to the breakdowns made in the normative dimension. This dimension is an important motivational force that has been overlooked and underutilized (McCormick and Donohue, 2016; Meyer and Parfyonova, 2010) and as McCormick and Donohue (2016) point out, they have been the object of conceptual reconfiguration over time (Wayne et al., 2009). In recent years, it has become a moral obligation (Meyer and Parfyonova, 2010). The second theoretical implication is that feelings promoted by the need for internal consistency and meeting responsibilities acquired (Moral Obligation dimension), based on cognitive dissonance theory of Festinger (1957), are stronger links with respect to the entity than those that may be generated by feelings of gratitude toward the organization, feelings that are reflected in the Gratitude dimension and based on the social exchange theory of Blau (1964), which does not show a sufficient level of variance and hence reliability.

Finally, in this study we have aimed at adapting organizational commitment to a specific field, and one which is as peculiar as voluntary organizations. The main theoretical contribution is, regarding NPOs, the most significant links to people who work in an NPO are those that are related to affective ties. In conclusion, this theoretical implication is aligned with motivational theory on intrinsic/extrinsic motivational factors and contribute to demonstrate how intrinsic motivations are more relevant than extrinsic motivations.

### Practical implications

Research on commitment in NPOs underlines the role that management and human resources practices can play in fostering employee commitment (Cunningham, 2001; Alatrista and Arrowsmith, 2004). To undertake such research it is essential to identify the factors that promote the commitment and retention of NPO employees (McCormick and Donohue, 2016).

The first managerial implication is the individual's emotional bonds to the organization, defined as affective commitment, which are of great importance to NPOs. Employees and volunteers of NPOs are highly sensitive to the organization's mission and values and they strongly identify with the organization's social mission. NPO internal consumers need these affective bonds to feel committed to the organization and to bring the best to the organization (Ohana and Meyer, 2016) and these bonds influence motivation (Somers, 2010; Cohen, 2011). In considering individual links, this commitment is key to attitudes and behaviors, including higher performance, organizational citizenship behavior, as well as lower levels of turnover and absenteeism (Cunningham, 2001; Meyer et al., 2002; Ridder and McCandless, 2010). Strong employee engagement with values, missions, and goals is therefore essential to organizational success and organizational survival (Ridder and McCandless, 2010). Incorporated into the stated mission, organizational values provide guidance and justification for the decisions and behavior of members of the organization (O'Reilly et al., 1991; Lawrence and Lawrence, 2009). In this sense, in accordance with our results, the main implication for managers is that they have to declare publicly and clearly the organization's mission and values, and this declaration is a key factor in addressing the following:

Regarding volunteer engagement, this is the main aspect with which to attract and retain motivated volunteers because those volunteers feel that they identify with the organizational mission and values. Often, volunteers are motivated to join organizations on the basis of the compatibility of their individual beliefs and values with the organizational values that are adopted (Amos and Weathington, 2008; Van Vuuren et al., 2008).

Motivational aspect for employees: Non-profit employees often feel they are underpaid (Light, 2003; Kim and Lee, 2007; Handy et al., 2008) but they may be willing to sacrifice some money in order to serve a cause or specific social mission (Ohana and Meyer, 2016). In this way, in a context of scarce financial resources, our second recommendation is that human resources managers should consider the definition of job position and must establish mechanisms to promote appropriate activities in considering how they contribute to the mission of the organization. This is more effective than other retention systems such as reward systems (continuance commitment).

It is thus crucial to stress those work experiences that contribute to the feeling of belonging, and to develop an organizational culture based on common values and goals rather than on economic rewards, which can be impractical and unusual in most organizations. There is also evidence of links related to feelings of obligation and normative commitment, which have come to be associated with an accountability and a greater control of one's own activity that promotes long-lasting behaviors, although concerning this type of relationship there is little empirical support that backs previous results of our work. Therefore, we consider it important to deepen the knowledge of elements linking people with the organization in the context of NPOs and the relationship among them.

### Limitations and future research lines

Although this work contributes to a better understanding of the nature of links between individuals and NPOs, it has also some limitations. The main limitation of this research is the size of the sample. In future works, it should be interesting to use larger and representative samples to deepen research into the differences between employees and volunteers. Although, the internal meaning of each dimension is still an open debate, and is necessary to clarify the relation between Affective and Normative dimensions, another future research could aim to analyze the relations of each dimension with other variables (e.g., satisfaction, social performance, organization culture) and their effects on them over time. We hope new works will explore these future research lines because are essential to develop mutually beneficial and satisfactory relationships between organization and person.

## Author contributions

The three authors have equally participated in literature review, data analysis and writing of the paper. All authors listed have made a substantial, direct and intellectual contribution to the work, and approved it for publication.

### Conflict of interest statement

The authors declare that the research was conducted in the absence of any commercial or financial relationships that could be construed as a potential conflict of interest. The reviewers JL, SG and handling Editor declared their shared affiliation, and the handling Editor states that the process nevertheless met the standards of a fair and objective review.
